# A novel 3D biofabrication strategy to improve cell proliferation and differentiation of human Wharton’s jelly mesenchymal stromal cells for cell therapy and tissue engineering

**DOI:** 10.3389/fbioe.2023.1235161

**Published:** 2023-08-10

**Authors:** Cristina Blanco-Elices, Roke Iñaki Oruezabal, David Sánchez-Porras, Jesús Chato-Astrain, Fernando Campos, Miguel Alaminos, Ingrid Garzón, Antonio Campos

**Affiliations:** ^1^ Tissue Engineering Group, Department of Histology, Faculty of Medicine, Universidad de Granada, Granada, Spain; ^2^ Instituto de Investigación Biosanitaria ibs.GRANADA, Granada, Spain; ^3^ Centro Nacional de Investigaciones Oncológicas (CNIO), Madrid, Spain

**Keywords:** cell culture, three-dimensional, biofabrication, human Wharton’s jelly mesenchymal stromal cells, cell therapy, tissue engineering

## Abstract

**Purpose:** Obtaining sufficient numbers of cells in a short time is a major goal of cell culturing in cell therapy and tissue engineering. However, current bidimensional (2D) culture methods are associated to several limitations, including low efficiency and the loss of key cell differentiation markers on cultured cells.

**Methods:** In the present work, we have designed a novel biofabrication method based on a three-dimensional (3D) culture system (FIBRIAGAR-3D). Human Wharton’s jelly mesenchymal stromal cells (HWJSC) were cultured in 3D using 100%, 75%, 50%, and 25% concentrations of fibrin-agarose biomaterials (FA100, FA75, FA50 and FA25 group) and compared with control cells cultured using classical 2D systems (CTR-2D).

**Results:** Our results showed a significant increase in the number of cells generated after 7 days of culture, with cells displaying numerous expansions towards the biomaterial, and a significant overexpression of the cell proliferation marker KI67 was found for the FA75 and FA100 groups. TUNEL and qRT-PCR analyses demonstrated that the use of FIBRIAGAR-3D was not associated with an induction of apoptosis by cultured cells. Instead, the 3D system retained the expression of typical phenotypic markers of HWJSC, including CD73, CD90, CD105, NANOG and OCT4, and biosynthesis markers such as types-I and IV collagens, with significant increase of some of these markers, especially in the FA100 group. Finally, our analysis of 8 cell signaling molecules revealed a significant decrease of GM-CSF, IFN-g, IL2, IL4, IL6, IL8, and TNFα, suggesting that the 3D culture system did not induce the expression of pro-inflammatory molecules.

**Conclusion:** These results confirm the usefulness of FIBRIAGAR-3D culture systems to increase cell proliferation without altering cell phenotype of immunogenicity and opens the door to the possibility of using this novel biofabrication method in cell therapy and tissue engineering of the human cornea, oral mucosa, skin, urethra, among other structures.

## 1 Introduction

The recent development of advanced therapy medicinal products allowed the generation of novel therapeutic products based on human cells (Olry de Labry-Lima et al., 2023). In general, cell therapy and tissue engineering use living cells that are initially obtained from tissue biopsies and subsequently expanded to obtain sufficient numbers of cells (Ortiz-Arrabal et al., 2022). Although cell culture methods have been improved during the last years, most methods are still subjected to important limitations regarding their efficiency, the time required to obtain abundant cell populations and the phenotypic changes that most primary cell cultures experience after *ex vivo* sequential passaging (Yan et al., 2014).

Several types of cells have been used in cell therapy and tissue engineering. Human Wharton’s jelly mesenchymal stromal cells (HWJSC) have numerous advantages as compared to other cell types, including high proliferation and differentiation capabilities and low immunogenicity when used *in vivo* (Garzon et al., 2020; Pochon et al., 2022). In fact, these cells express different cell markers of MSC in the umbilical cord before and after *ex vivo* isolation (Garzón et al., 2014; Sánchez-Porras et al., 2023). Specifically, HWJSC typically fulfill the minimal criteria of human mesenchymal stromal cells (Dominici et al., 2006), such as CD90, CD105, CD73 and vimentin, while not expressing CD34, CD45, CD14, CD19 or Major Histocompatibility Complex, Class II, DR (HLA-DR) (Bharti et al., 2018), but also express several markers of pluripotency, such as the homeobox transcription factor NANOG and the octamer-binding transcription factor 4 (OCT-4) (Musiał-Wysocka et al., 2019). As some of these markers are most likely associated with closed features of HWJSC used in cell therapy and tissue engineering, retaining the expression of these markers should be an objective of culture systems intended for clinical application of HWJSC.

Most available MSC-related studies have been conducted using bidimensional (2D) cell cultures established on culture surfaces. Although these systems allow cell expansion, development of more efficient culture protocols providing higher cell expansion efficiency is needed. In fact, it has been demonstrated that MSC tend to diminish their proliferation potential and their differentiation capability when cultured on 2D systems (Yan et al., 2014; McKee and Chaudhry, 2017), with a significant reduction in the expression of some of the typical markers of pluripotency expressed by MSC (Hess et al., 2017; Mousavifard et al., 2020; Thakur et al., 2022).

It is well known that human cell physiology is strictly dependent on the surrounding extracellular matrix (ECM), and cell survival and function is often mediated through cell-cell interactions, but also, through cell-ECM interactions (Habanjar et al., 2021; Sung et al., 2023). In the human tissue, cell adhesion, proliferation, survival and differentiation are directly controlled and regulated by three-dimensional (3D) cell-ECM interactions mediated by transmembrane cell receptors and ECM integrins (Brizzi et al., 2012). The lack of these important clues and interactions in traditional 2D culture methods could contribute to explain why MSC cultured in culture plates tend to lose proliferation and differentiation potential (Mousavifard et al., 2020).

In this context, recent developments focused on optimization of cell culture protocols using 3D culture systems able to reproduce the native niche of cultured cells (Bruschi et al., 2022). Among others, the use of biocompatible biomaterials allowing the generation of complex tissues and organs by tissue engineering (Ionescu et al., 2020; González-Gallardo et al., 2023), could contribute to reproduce the 3D microarchitecture of native cells. Although different biomaterials have been proposed for this purpose, such as collagen (Song et al., 2023), alginate (Jahanbakhsh et al., 2020) and fibrinogen (Simaan-Yameen et al., 2023), among others, the ideal biomaterial to be used in 3D culturing of human MSC has not been determined to the date.

The potential of a novel 3D culture platform called FIBRIAGAR-3D was evaluated in the present work. This system is based on different concentrations of fibrin-agarose biomaterials and its use could contribute to increase cell proliferation of HWJSC without altering the differentiation capability and immunogenicity of these cells. Results could contribute to improve current cell culture methods for use in cell therapy and tissue engineering protocols.

## 2 Materials and methods

### 2.1 Establishment of primary cell cultures of human Wharton’s jelly stromal cells (HWJSC)

Primary cell cultures of Human Wharton’s Jelly Stromal Cells (HWJSC) were established from umbilical cords obtained from the Human Tissue Biobank of the Andalusian Public Health System. Small fragments were surgically obtained from the Wharton’s jelly and digested in 2 mg/mL solution of *Clostridium hystoliticum* type I collagenase (Thermo Fisher Scientific-Gibco, Waltham, Massachusetts, United States) at 37°C for 6 h. Cells were then harvested by centrifugation and cultured in culture flasks using DMEM-Advanced culture medium (Thermo Fisher Scientific-Gibco) supplemented with 10% fetal bovine serum (Merck) until subconfluence. Cells were then enzymatically detached using a commercial detachment solution containing 0.5 g/L of trypsin and 0.2 g/L of EDTA (Merck, Darmstadt, Germany) and used in the studies described below.

This study was conducted according to the guidelines of the Declaration of Helsinki and approved by the Institutional Ethics Committee for Biomedical Research in Andalusia (*Comité Coordinador de Ética de la Investigación Biomédica de Andalucía*), protocol code 0116-N-19, date of approval 29th, may, 2019.

### 2.2 Generation of FIBRIAGAR-3D culture platforms

In the first place, we generated fibrin-agarose biomaterials following previously described protocols (Blanco-Elices et al., 2023). Briefly, to generate 1 mL of biomaterial, we mixed 760 µL of human plasma with 15 µL of tranexamic acid (Amchafibrin 5 mg/mL, MEDA Pharma SL, Madrid, Spain), 100 µL of a 2% solution of agarose in PBS (Merck), 50 µL of a 1% solution of CaCl_2_ (Merck), and 75 µL of DMEM-Advanced culture medium (Thermo Fisher Scientific-Gibco). 150,000 detached HWJSC were added at the last step. The human plasma used in this work was obtained from healthy blood donors and contained a physiological amount of fibrinogen, as previously described for the generation of human bioartificial tissues by tissue engineering for clinical use (González-Gallardo et al., 2023). To evaluate the effects of the FIBRIAGAR-3D cell culture platform, HWJSC were cultured with different concentrations of a fibrin-agarose biomaterial. For the platform consisting in a 100% concentration of the biomaterial (FA100 group), the mixture described here was directly aliquoted in 24-well culture plates (Sarstedt, Nümbrecht, Germany) and allowed to jellify at 37°C in a cell culture incubator. For the platform with 75% of biomaterial (FA75 group), the mixture was diluted at a 3:1 proportion in DMEM-Advanced culture medium before adding the WHJSC, aliquoting the mixture and allowing the biomaterial to jellify. In the case of the 50% group (FA50), the biomaterial was diluted 1:1 in DMEM-Advanced, whereas the 25% group (FA25) was diluted at a 1:3 proportion in DMEM-Advanced. In all cases, the same culture plates and jellification conditions were used, and the same number of cells was used in all groups. As a control (CTR-2D group), 150,000 detached HWJSC were subcultured directly on 24-well culture plates without any biomaterial, resembling traditional 2D culture methods. Cells of the CTR-2D groups and jellified biomaterials in the FIBRIAGAR-3D groups were covered by DMEM-Advanced culture medium with 10% fetal bovine serum. Results were analyzed after 7 days of culture at 37°C with 5% CO_2_ in a cell incubator.

### 2.3 Histological and immunohistochemical analyses

For histological analysis, cells cultured using the different concentrations of FIBRIAGAR-3D and CTR-2D were fixed in 4% formaldehyde, washed, dehydrated, cleared, and embedded in paraffin. Tissue sections were obtained and stained with hematoxylin and eosin (HE) following routine histological methods, and the morphology and histological conformation of the cells corresponding to each study group was evaluated. In addition, histological sections stained with HE were used to quantify the number of cells found in each study group. In each sample, the number of stained cells was determined per 2000 μm^2^ of area. A total of 10 independent measures were obtained per study group (n = 10).

Immunohistochemical analyses were performed to identify the presence of relevant markers and tissue components in each study group. Immunohistochemistry using the marker of proliferation KI67 (Thermo Fisher Scientific, Waltham, Massachusetts, United States, PA1-38032, dilution 1:100) was performed. To confirm the mesenchymal nature of the cultured cells, vimentin expression was assessed. MSC phenotype was evaluated by immunohistochemistry for CD73 (Abcam, Cambridge, United Kingdom, ab175396, dilution 1:200), CD90 (Abcam, ab92574, dilution 1:25) and CD105 (Abcam, ab169545, dilution 1:200) cell markers. Finally, the biosynthetic activity of HWJSC was investigated in the different study groups by immunohistochemistry for type-I collagen (COL-I; OriGene Technologies GmbH, Herford, Germany, R1038, dilution 1:500) and type-IV collagen (COL-IV; Master Diagnostica, Granada, Spain, MAD-000733QD, ready to use). In brief, tissue sections were obtained, deparaffinized and rehydrated, and treated with an antigen retrieval solution for 20 min (PanReac, Barcelona, Spain). Samples were incubated in 3% H_2_O_2_ for 10 min to quench endogenous peroxidase activity, and unspecific staining was blocked with 2.5% horse serum and casein (Vector Laboratories, Burlingame, CA, United States) for 20 min. Then, samples were incubated overnight with primary antibodies at 4°C, washed in PBS and incubated with secondary antibodies for 1 h at RT. The complex epitope-antibody was detected using 3,3-diaminobenzidine -DAB- (Vector Laboratories), and tissue sections were counterstained for 20 s using Harry’s hematoxylin (Gibco-Thermo Fisher Scientific, Waltham, MA, United States). Native human controls and study samples in which the staining method was performed without primary antibody were used as technical controls (Supplementary Figure S2). In all cases, images were obtained using a Pannoramic^®^ DESK II DW scanner (3D Histotech, Budapest, Hungary). Quantitative analysis of the immunohistochemical images was performed by evaluating the number of cells showing positive and negative immunostaining signal for each marker in a prefixed square area of 350 × 350 μm^2^, with further calculation of the percentage of positive cells. A total of 10 independent measures were obtained per study group (*n* = 10).

### 2.4 Analysis of cell death using TUNEL assays

Terminal deoxynucleotidyl transferase (TdT) dUTP Nick-End Labeling (TUNEL) assays were used to identify cells undergoing apoptotic cell death in each study group using a DeadEnd™ Fluorometric TUNEL System (Promega, Madison, Wisconsin), following the instructions provided by the manufacturer. In brief, tissue sections were dewaxed and treated with a 20 μg/mL solution of proteinase K for 5 min at room temperature, washed in PBS and incubated in the TUNEL reaction solution of the kit for 1 h in the dark at 37°C. Samples were then washed in PBS, dried and mounted using VECTASHIELD^®^ + 4′,6-diamidino-2-phenylindole (DAPI) (Vector Laboratories), and images were obtained using a Nikon Eclipse i90 fluorescence microscope. Quantitative analyses were carried out by counting the number of cells undergoing apoptosis (stained in green) and normal cells (stained in blue) in each image of 25,000 μm^2^, and the percentage of apoptotic cells was calculated. Samples used as technical controls are shown in Supplementary Figure S2. Three independent samples were analyzed per group of study (*n* = 3).

### 2.5 Gene expression analysis

Gene expression was evaluated at the mRNA level using quantitative real-time RT-PCR (qRT-PCR). First, total RNA was extracted from each study group using a Qiagen RNeasy Mini Kit (Qiagen, Mississauga, ON, Canada). Then, RNA was retrotranscribed to cDNA with an iScript Advanced cDNA Synthesis Kit containing a retrotranscriptase (Bio-Rad Laboratories, Hercules, CA, United States), and this cDNA was used to amplify and quantify the different genes analyzed here. In the present work, we analyzed the expression of genes playing a role in cell proliferation, such as the proliferating cell nuclear antigen (*PCNA*); migration, such as cortactin (*CTTN*); apoptosis, including caspase 8 (*CASP8*), B-cell lymphoma 2 apoptosis regulator (*BCL2*) and X-linked inhibitor of apoptosis (*XIAP*); and stemness phenotype, including *NANOG*, *OCT4*, SRY-box transcription factor 2 (*SOX2*) and *CD45* using a Prime-PCR system customized PCR plate (Bio-Rad Laboratories), and glyceraldehyde-3-phosphate dehydrogenase (*GAPDH*) was used as a control housekeeping gene. Briefly, equal amounts of cDNA were mixed with SsoAdvanced Universal SYBR^®^ Green Supermix (Bio-Rad Laboratories) and the PCR reaction was carried out using a Bio-Rad CFX Connect 96 thermocycler. Results were normalized to GAPDH expression. 6 independent samples were analyzed per study group (*n* = 6).

### 2.6 Quantification of cytokine and chemokine cell signaling molecules

To evaluate the potential effects of 3D culturing on the immunogenicity of HWJSC, we assessed several bioactive cell signaling molecules secreted to the culture medium using the Bio-Plex Pro Human Cytokine 8-plex Assay #M50000007A (Bio-Rad Laboratories). This system was able to quantitatively evaluate 8 bioactive molecules, including granulocyte-macrophage colony-stimulating factor (GM-CSF), interferon gamma (INF-g), interleukins 2, 4, 6, 8, and 10 (IL2, IL4, IL6, IL8 and IL-10) and tumor necrosis factor alpha (TNFα). Supernatant culture medium was harvested from each study group (CTR-2D, FA25, FA50, FA75, and FA100) and diluted 4-fold with diluent buffer, as suggested by the manufacturer. These samples were then incubated with the antibody-bead conjugates in 96-well microplates and at 20°C–25°C for 1 h with shaking, followed by incubation with a biotin-labeled antibody at 20°C–25°C for 30 min and streptavidin-phycoerythrin conjugates at 20°C–25°C for 10 min (both, after washing and shaking). Finally, the assay buffer was added, and fluorescence for the Eight analytes was measured using an automatic immunoassay analyzer (Bio-Plex^®^ 200 System; Bio-Rad Laboratories). Finally, the cytokine concentration was calculated using standard curves. Eight independent samples were analyzed per study group (*n* = 8).

### 2.7 Statistical analysis

For each study group, and for each analysis method, average values and standard deviation were first calculated. Then, each distribution was analyzed for normality using the Shapiro-Wilk statistical test. As we demonstrated that most variables were not normally distributed, and therefore, did not fulfill the criteria for parametric analysis, non-parametric statistical tests were then used. To compare the number of cells obtained in each study group, the percentage of cells showing positive immunohistochemical signal for KI67, VIMENTIN, CD73, CD90, CD105, COL-I, and COL-IV, and for the TUNEL and cell signaling molecules assay, we used Mann-Whitney *U* exact test. For the gene expression analysis, we first determined the fold-change relative expression of each experimental group compared with the CTR-2D group (considered as 1). Then the statistical significance p-value was calculated for each experimental group compared to CTR-2D using Mann-Whitney *U* exact test.

All statistical tests were performed using Excel Real Statistics (http://www.real-statistics.com) (Dr. Charles Zaiontz, Purdue University, West Lafayette, IN, United States). The significance level was set at 5% for the two-tail analyses.

## 3 Results

### 3.1 Histological analysis of cells cultured in the different conditions

**FIGURE 1:**
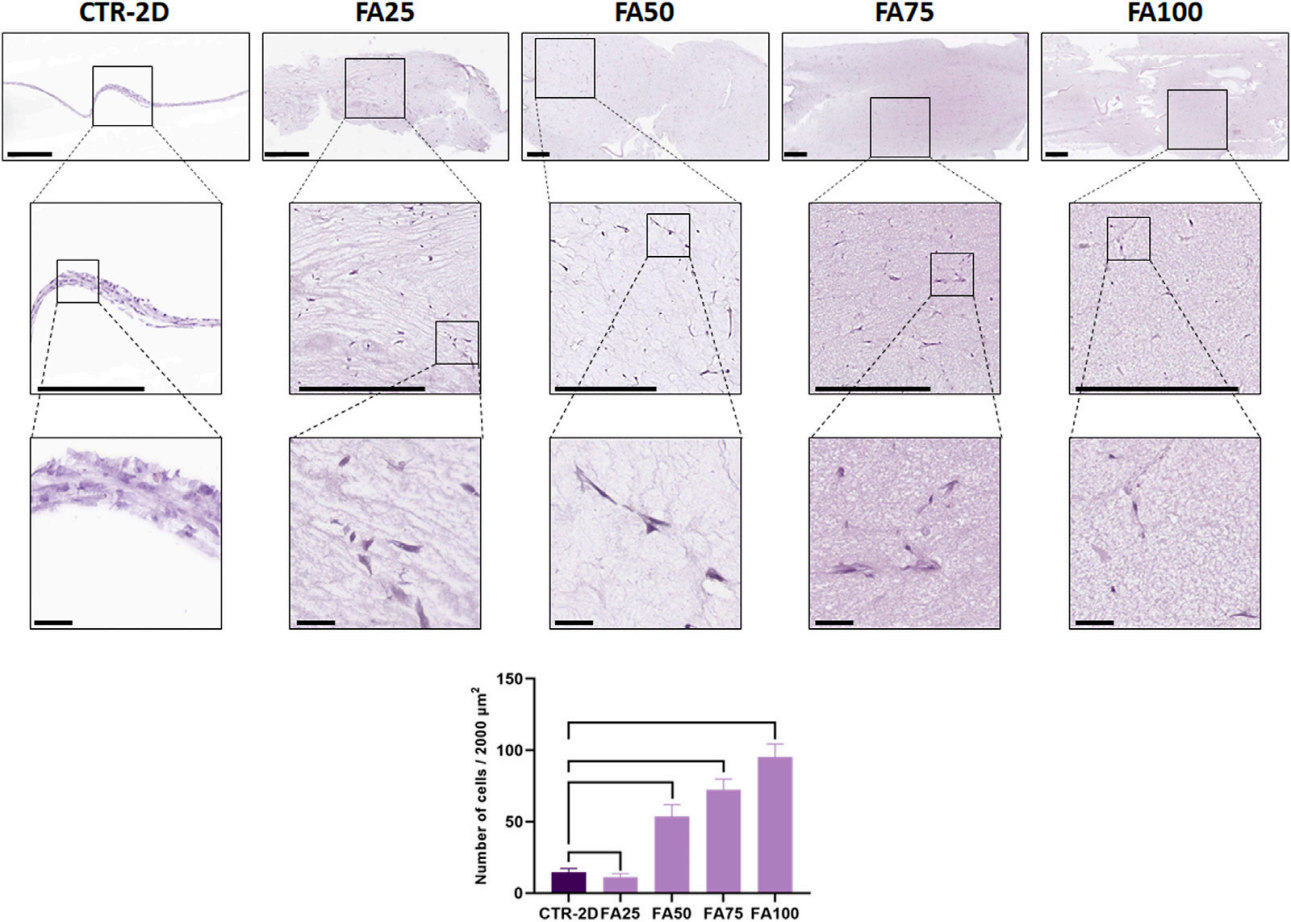
Histological analysis of HWJSC cultured on culture plates (CTR-2D) and in the 25%, 50%, 75% and 100% concentrations of FIBRAGAR-3D (FA25, FA50, FA75, and FA100, respectively). Tissue sections were stained with hematoxylin-eosin (HE), and different magnification images are shown for each culture condition. Scale bars: 500 µm for the top and medium rows and 50 µm for the bottom row. The histogram shows the average and standard deviation number of cells found in 2000 µm of culture surface for each study group. Black lines connecting two groups indicate that the differences are statistically significant.

In the first place, we analyzed the morphology and number of HWJSC cultured in the FIBRIAGAR-3D system on tissue sections stained with HE. Results showed that cells were able to grow within the FA biomaterial, showing a spindle shape or a star-like morphology, with cell expansions to all the spatial directions (Figure 1). In contrast, cells cultured in CTR-2D tended to be flat and elongated. No morphological signs of apoptosis, necrosis or other type of cell alteration was detected in any of the study groups.

### 3.2 Analysis of cell proliferation in each study group

Quantification of the number of cells found in each study group after the follow-up period (Figure 1) showed significant differences among groups. First, we found that the CTR-2D samples contained 14.6 ± 2.62 cells in 2000 µm of culture surface, whereas cells cultured in FA25 showed significantly lower number of cells (11.11 ± 2.51 cells, *p* = 0.0101). However, cells cultured in FA50, FA75 and FA100 contained significantly higher number of cells (53.6 ± 8.32, 72.4 ± 7.41, and 95.3 ± 9.08 cells, respectively) than CTR-2D, with differences being statistically significant (*p* < 0.0001 for all comparisons).

At the mRNA level, our analysis of PCNA gene expression revealed some differences between the cells cultured on culture flasks in the CTR-2D group and cells cultured using the FIBRIAGAR-3D system, although these differences were not statistically significant. As shown in Table 1, expression of this gene in cells cultured in FA100 was 2.74 ± 1.45 times higher than that of CTR-2D. The same pattern was found for the expression of CTTN, with more than 2-fold expression in FA100, although differences were not statistically significant.

**TABLE 1:** Expression of the different genes analyzed in this work using qRT-PCR on HWJSC cultured on culture plates (CTR-2D) and in the 25%, 50%, 75%, and 100% concentrations of FIBRAGAR-3D (FA25, FA50, FA75, and FA100, respectively).

	CTR-2D	FA25	FA50	FA75	FA100
GAPDH	1 ± 0	1 ± 0	1 ± 0	1 ± 0	1 ± 0
PCNA	1 ± 1.18	0.19 ± 0.08	1.31 ± 0.8	1.25 ± 1.47	2.74 ± 1.45
CTTN	1 ± 1.24	0.08 ± 0.04	1.02 ± 0.95	1.78 ± 1.98	2.88 ± 1.79
CASP8	1 ± 1.43	0.57 ± 1.12	0.82 ± 0.33	2.22 ± 2.87	2.24 ± 1.16
BCL2	1 ± 1.1	0.86 ± 1.01	1.99 ± 2.93	1.03 ± 0.61	1.11 ± 1.02
XIAP	1 ± 1.43	0.88 ± 1.35	1.24 ± 1.25	1.9 ± 2.69	0.15 ± 0.09
NANOG	1 ± 1.43	0.01 ± 0	2.68 ± 2.82	6.08 ± 7.34	6.77 ± 3.72*
OCT4	1 ± 1.02	0.01 ± 0	1.61 ± 1.03	2.44 ± 1.97	3.18 ± 1.77*
SOX2	1 ± 1.34	0.07 ± 0.12	0.24 ± 0.15	0.54 ± 0.75	1.41 ± 1.36
CD45	1 ± 1.72	0.01 ± 0	1.54 ± 0.79	4.33 ± 4.09	2.83 ± 0.94

For each type of sample, the fold-change relative expression is shown as compared to results obtained in the CTR-2D group (considered as 1), after GAPDH normalization.

Results are shown as averages ± standard deviations.

Results showing statistically significant differences with CTR-2D are labeled with asterisks (*).

**FIGURE 2:**
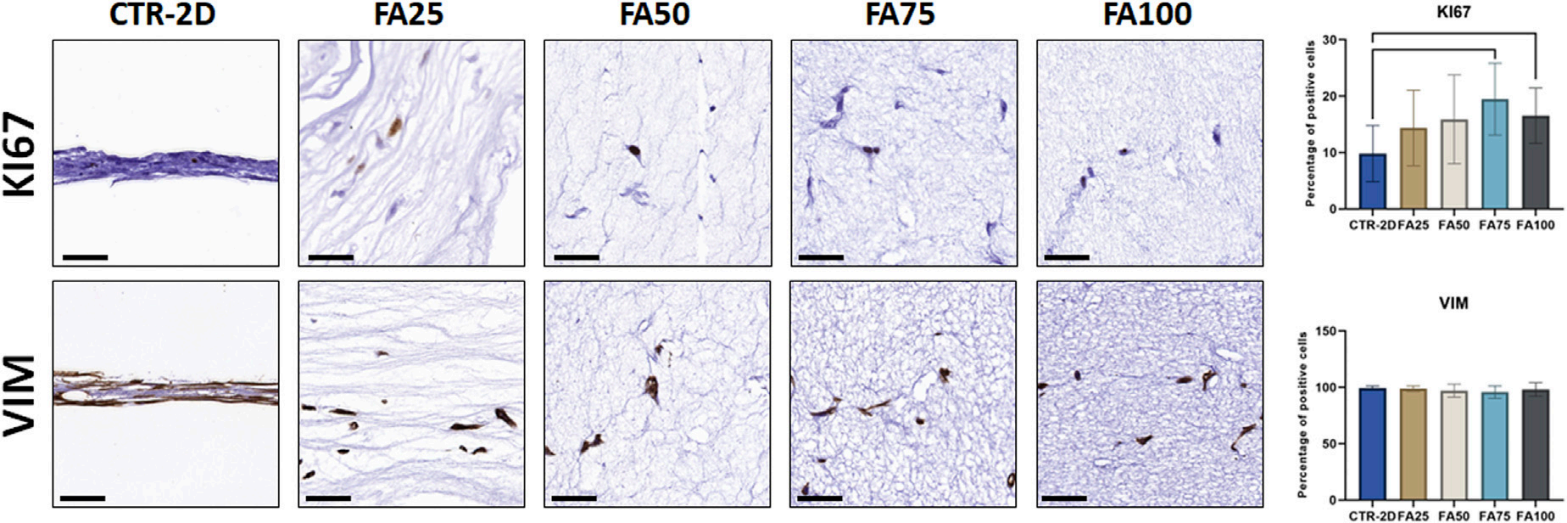
Immunohistochemical analysis of KI67 and vimentin (VIM) expression in HWJSC cultured on culture plates (CTR-2D) and in the 25%, 50%, 75% and 100% concentrations of FIBRAGAR-3D (FA25, FA50, FA75, and FA100, respectively). Scale bars: 50 µm. The histograms show the average and standard deviation percentage of positive cells for each study group. Black lines connecting two groups indicate that the differences are statistically significant.

Immunohistochemical analysis of the proliferation marker KI67 on cells cultured using the different culture systems described in this work (Figure 2) found that the percentage of KI67-positive cells was significantly higher in FA75 and FA100 as compared to the CTR-2D group (*p* = 0.0039 and 0.0266, respectively). No differences were found between FA25 or FA50 and CTR-2D (Table 2). On the other hand, the analysis of vimentin showed that more than 95% of the cells found in all study groups were positive for this marker, without statistical differences among groups (Figure 2; Table 2).

**TABLE 2:** Percentage of cells showing positive expression of the different immunohistochemical markers analyzed in this work in HWJSC cultured on culture plates (CTR-2D) and in the 25%, 50%, 75%, and 100% concentrations of FIBRAGAR-3D (FA25, FA50, FA75, and FA100, respectively).

	KI67	VIMENTIN	CD73	CD90	CD105	COL-I	COL-IV
CTR-2D	9.78 ± 4.97	99.4 ± 1.8	100 ± 0	74.84 ± 4.21	78.55 ± 8.22	79.89 ± 12.23	99.85 ± 0.45
FA25	14.35 ± 6.68	98.86 ± 2.29	100 ± 0	66.02 ± 16.67	95.7 ± 5.42	88.66 ± 10.65	97.56 ± 4.91
FA50	15.88 ± 7.88	97.17 ± 5.68	100 ± 0	64.29 ± 15.93	94.16 ± 4.53	100 ± 0	98.75 ± 3.75
FA75	19.47 ± 6.35	95.8 ± 5.46	100 ± 0	69.67 ± 4.48	98.33 ± 5	100 ± 0	96.46 ± 5.9
FA100	16.52 ± 4.9	98 ± 6	100 ± 0	67.51 ± 15.12	95.14 ± 8.19	100 ± 0	86.9 ± 22.4
CTR-2D vs. FA25	0.1728	0.7959	0.9705	0.0927	0.0001*	0.1051	0.6842
CTR-2D vs. FA50	0.0892	0.6842	0.9705	0.1139	0.0001*	0.0002*	0.9705
CTR-2D vs. FA75	0.0039*	0.2743	0.9705	0.0592	0.0001*	0.0002*	0.3930
CTR-2D vs. FA100	0.0266*	0.9705	0.9705	0.1011	0.0021*	0.0002*	0.2176
FA25 vs. FA50	0.8286	0.9118	0.9705	0.9314	0.7197	0.0068*	0.7394
FA25 vs. FA75	0.1011	0.4082	0.9705	0.2973	0.3527	0.0068*	0.7394
FA25 vs. FA100	0.3823	0.7959	0.9705	0.8421	0.8286	0.0068*	0.3150
FA50 vs. FA75	0.1655	0.7618	0.9705	0.4363	0.0653	0.9705	0.4813
FA50 vs. FA100	0.5726	0.7959	0.9705	0.7197	0.3704	0.9705	0.2176
FA75 vs. FA100	0.2743	0.4082	0.9705	0.4002	0.3599	0.9705	0.4813

Average and standard deviation values are shown for each marker and each study group.

The last rows show the statistical p values for the comparison of two specific groups of samples.

Significant p values are labeled with asterisks (*).

### 3.3 Analysis of HWJSC cell death induced by the FIBRIAGAR-3D culture system

First, we quantified the mRNA corresponding to the pro-apoptotic gene CASP8, and we found non-significant differences between cells cultured in FIBRIAGAR-3D (FA25, FA50, FA75 and FA100) and cells corresponding to the CTR-2D group (Table 1). Similarly, the analysis of the anti-apoptotic genes BCL2 and XIAP resulted in non-significant differences with cells cultured on 2D surfaces.

Then, we carried out TUNEL analyses to identify cells undergoing apoptosis in each study group (Supplementary Figure S1). Results showed that the percentage of cells showing positive TUNEL staining was 11.46% ± 9.98% in the CTR-2D group, with non-significant differences in the different FIBRIAGAR-3D concentrations (3.67% ± 7.77% for FA25, 10% ± 16.1% for FA50, 5% ± 10.54% for FA75% and 10.29% ± 13.17% for FA100; *p* > 0.05 for all comparisons).

### 3.4 Analysis of HWJSC stemness phenotype

In order to evaluate the cell phenotype of WHJSC cultured in FIBRIAGAR-3D, we first analyzed the expression of several genes playing an important role in cell stemness. As shown in Table 1, our results showed that cells corresponding to the FA100 group significantly overexpressed NANOG and OCT4, as compared to CTR-2D (*p* = 0.0087 and 0.0260, respectively). Differences were non-significant for SOX2 and for the control gene CD45.

**FIGURE 3:**
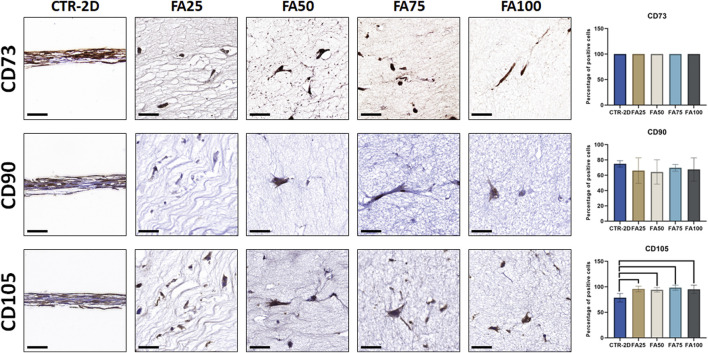
Immunohistochemical analysis of CD73, CD90, and CD105 expression in HWJSC cultured on culture plates (CTR-2D) and in the 25%, 50%, 75%, and 100% concentrations of FIBRAGAR-3D (FA25, FA50, FA75 and FA100, respectively). Scale bars: 50 µm. The histograms show the average and standard deviation percentage of positive cells for each study group. Black lines connecting two groups indicate that the differences are statistically significant.

In addition, we evaluated the expression of several cell-surface markers of cell stemness (Figure 3; Table 2), and we found that culturing the cells on the different concentrations of FIBRIAGAR-3D maintained the high expression of the markers CD73 and CD90 found in CTR-2D, with differences being non-significant. However, the 3D system was able to increase the percentage of cells showing positive expression of CD105 in FA25, FA50, FA75, and FA100, with more than 94% of positive cells in all these conditions, whereas 78.55% ± 8.22% of the cells cultured in 2D systems were positive for this marker. Differences between CTR-2D and the four concentrations of FIBRIAGAR-3D were statistically significant (*p* = 0.0001 for FA25, FA50 and FA75, and *p* = 0.0021 for FA100).

### 3.5 Evaluation of the collagen biosynthetic activity of HWJSC cultured in FIBRIAGAR-3D

**FIGURE 4:**
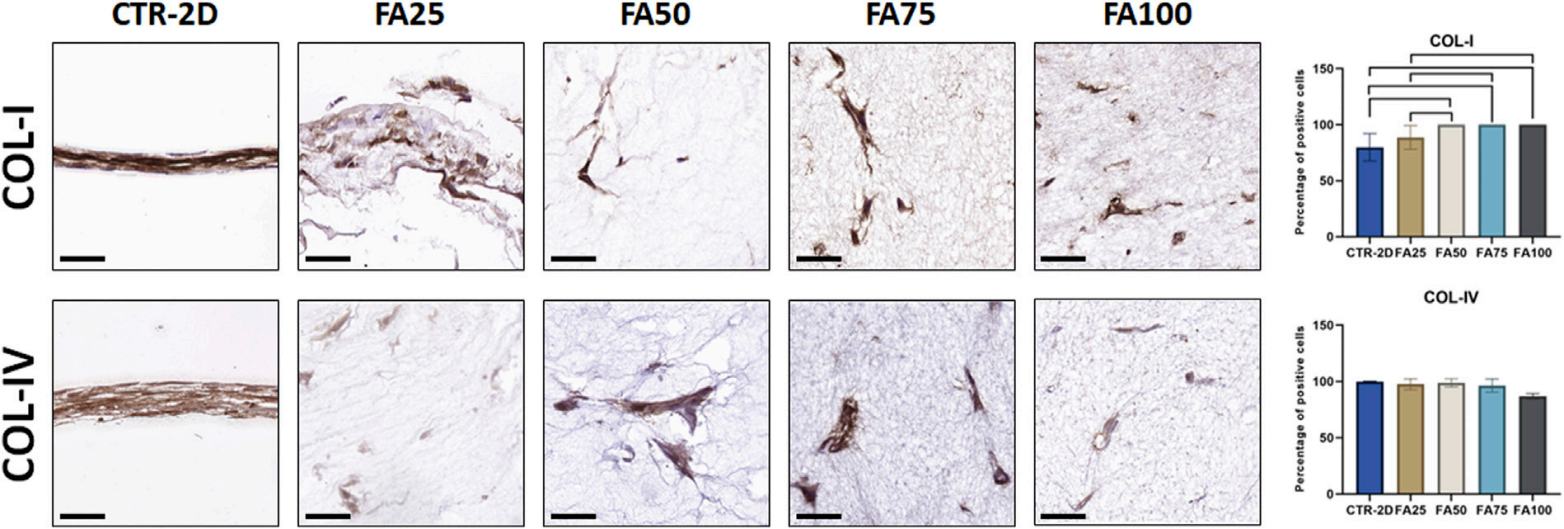
Immunohistochemical analysis of type-I collagen (COL-I) and type-IV collage (COL-IV) expression in HWJSC cultured on culture plates (CTR-2D) and in the 25%, 50%, 75%, and 100% concentrations of FIBRAGAR-3D (FA25, FA50, FA75, and FA100, respectively). Scale bars: 50 µm. The histograms show the average and standard deviation percentage of positive cells for each study group. Black lines connecting two groups indicate that the differences are statistically significant.

Synthesis of type-I collagen was assessed in cells cultured using the FIBRIAGAR-3D system and control cells cultured on 2D culture surfaces. As shown in Figure 4; Table 2, we found that 79.89% ± 12.23% of cells of the CTR-2D group showed positive expression of type-I collagen. However, HWJSC cultured in FA50, FA75 and FA100 showed a significantly higher percentage of positive cells that reached 100% of the analyzed cells (*p* = 0.0002 in the three cases). Regarding type-IV collagen, all conditions showed more than 85% of positive cells, with non-significant differences among groups Figure 3; Table 2.

### 3.6 Analysis of cytokines and chemokine cell signaling molecules in cells cultured with FIBRIAGAR-3D

To determine the putative immunogenic effects of cells cultured in the 3D system, we analyzed 8 relevant cell signaling molecules secreted by the cultured cells. As shown in Table 3, cultured cells synthetized several signaling molecules, especially IL6, IL8 and GM-CSF, with differences among study groups. In general, culture of the HWJSC using FA50 tended to show increased amounts of the 8 molecules analyzed, whereas FA100 tended to decrease the concentration of these molecules. Specifically, FA100 was associated with a statistically significant decrease of GM-CSF, IFN-g, IL2, IL4, IL6, IL8, and TNFα, although non-significant differences were detected for IL10.

**TABLE 3:** Quantitative analysis of 8 relevant cell signaling molecules generated by HWJSC cultured on culture plates (CTR-2D) and in the 25%, 50%, 75% and 100% concentrations of FIBRAGAR-3D (FA25, FA50, FA75 and FA100, respectively) using Bio-Plex Human Cytokine 8-plex assays.

	GM-CSF	IFN-g	IL2	IL4	IL6	IL8	IL10	TNFα
CTR-2D	124.09 ± 186.63	22.86 ± 1.59	9.8 ± 1.11	32.8 ± 2.84	9717.04 ± 457.77	2720.79 ± 334.4	0.96 ± 0.59	19.94 ± 1.34
FA25	88.39 ± 62.45	22.02 ± 3.23	10.92 ± 1.29	26.9 ± 14.63	9464.26 ± 1121.2	4003.61 ± 272.37	1.51 ± 0.25	21.88 ± 1.98
FA50	109.92 ± 50.65	38.2 ± 5.31	15.83 ± 2.54	62.19 ± 3.73	11894.36 ± 824.24	5643.1 ± 596.52	1.04 ± 1.34	32.2 ± 5.32
FA75	29.68 ± 25.96	25.1 ± 5.91	12.53 ± 5.21	21.15 ± 5.78	9205.61 ± 1852.64	3261.67 ± 404.19	1.63 ± 2.54	20.33 ± 12.15
FA100	3.38 ± 3.4	5.22 ± 3.51	1.84 ± 2.51	21.15 ± 5.34	4117.36 ± 1076.96	609.8 ± 243.41	0.58 ± 0.43	3.39 ± 2.5
CTR-2D vs. FA25	0.1049	0.3282	0.1049	0.1206	0.8785	0.0002*	0.0649	0.0939
CTR-2D vs. FA50	0.1206	0.0002*	0.0003*	0.0003*	0.0002*	0.0002*	0.6620	0.0006*
CTR-2D vs. FA75	0.7209	0.6454	0.3282	0.0006*	0.3282	0.0070*	0.8357	0.9551
CTR-2D vs. FA100	0.0002*	0.0002*	0.0003*	0.0003*	0.0002*	0.0002*	0.5728	0.0003*
FA25 vs. FA50	0.5358	0.0002*	0.0006*	0.0002*	0.0003*	0.0002*	0.1419	0.0003*
FA25 vs. FA75	0.0104*	0.2345	0.8785	0.7984	0.6454	0.0047*	0.1014	0.6454
FA25 vs. FA100	0.0002*	0.0002*	0.0002*	0.7984	0.0002*	0.0002*	0.0047*	0.0002*
FA50 vs. FA75	0.0037*	0.0019*	0.0541	0.0002*	0.0104*	0.0002*	0.6943	0.0721
FA50 vs. FA100	0.0003*	0.0002*	0.0003*	0.0002*	0.0002*	0.0002*	0.9591	0.0003*
FA75 vs. FA100	0.0104*	0.0002*	0.0003*	0.9591	0.0002*	0.0002*	0.7789	0.0104*

For each molecule, average and standard deviation values are shown for each study group.

The last rows show the statistical p values for the comparison of two specific groups of samples.

Significant p values are labeled with asterisks (*).

## 4 Discussion

The search of novel and more efficient cell culture systems is a requirement of current research, and it has been proposed that 3D systems can simulate *in vivo* physiological environments more efficiently than 2D systems (Baruffaldi et al., 2021). In the present work, we evaluated the usefulness of FIBRIAGAR-3D culture systems to improve current cell culture methods applied to HWJSC.

It is well known that traditional cell isolation and culture techniques typically provide limited expansion, and long culture times are necessary to obtain large number of cells for therapeutic use (McKee and Chaudhry, 2017). For this reason, several alternative cell culture methods have been proposed, such as tissue explant, use of bioreactors or 3D culturing using different biomaterials (Hendijani, 2017; Scibona and Morbidelli, 2019). In this regard, we found that FIBRIAGAR-3D was able to increase the number of cells obtained at the end of the follow-up period, as compared to traditional cell culture methods, especially at the highest concentrations. Importantly, FA100 was able to increase the number of cells more than 6-fold, which is higher than the 1.6-fold reported for other biomaterial-based 3D systems such as Matrigel (Yan et al., 2014) or the 4-fold found for decellularized ECM (Lin et al., 2012). The higher number of cells was associated to increased cell proliferation, demonstrated by an increment in the expression of the cell proliferation-related protein KI67 and, partially of PCNA, confirming the potential of FIBRIAGAR-3D to improve cell proliferation.

When cells expanded in this system were evaluated histologically, we found that cell morphology was compatible with a normal cell population devoid of any detectable alterations, and kept their native expression of the MSC marker vimentin (Kuburich et al., 2022). In addition, cells cultured in FIBRIAGAR-3D had abundant expansions and spread in all spatial directions of the biomaterial. These results, along with the fact that these cells expressed increased amounts of vimentin and cortactin, suggest that the FIBRIAGAR-3D microenvironment promotes cell migration and 3D expansion within the biomaterial. Several works previously demonstrated that cortactin stimulates cell migration and cell attachment to the ECM mediated by cadherins and beta-catenins (van Rossum et al., 2006; Schnoor et al., 2018), whereas vimentin plays an important role in directing and controlling cell migration (Kuburich et al., 2022). In addition to this, our analysis of cell death revealed that cells cultured using the FIBRIAGAR-3D platform were not affected by apoptosis, suggesting that this technology is safe and did not affect cell viability, which is one of the main requirements of cells cultured for future clinical use (Cases-Perera et al., 2022). Future studies should be carried out to determine if cells cultured in FIBRIAGAR-3D may be associated with a process of senescence, and if this phenomenon could be reduced and controlled by this culture system as compared to 2D culturing. In this milieu, previous studies published by our group found that HWJSC tend to show senescence and decreased cell viability after sequential passaging and subculturing, with the fifth and sixth passages showing the highest viability levels (Garzón et al., 2012).

In addition to cell viability, culture systems should be able to support cell phenotype and function. In the case of the human MSC, cells should be able to express relevant markers of stemness and multipotentiality, such as CD73, CD90 and CD105, while not expressing other markers such as CD45 as previously established for this type of cells (Dominici et al., 2006; Bharti et al., 2018). In addition, the expanded differentiation potential of HWJSC to different types of cells (Garzon et al., 2020) has been demonstrated to be mediated by the expression of pluripotency markers, including NANOG, OCT-4, and SOX2 (Musiał-Wysocka et al., 2019). Although maintaining all these potency markers should be an objective of culture methods designed for HWJSC expansion, cells cultured using classical 2D culture systems typically tend to lose clonal and differentiation potential (McKee and Chaudhry, 2017), especially when cells are kept in culture for long periods of time (Martin-Piedra et al., 2014).

Our results showed that HWJSC cultured in FIBRIAGAR-3D significantly overexpressed CD105 at all concentrations, and NANOG and OCT4 at the highest concentration, as compared to cells cultured using 2D methods, and kept the expression of CD73, CD90 and SOX2. These results are in agreement with previous reports demonstrating that 3D culturing is able to enhance pluripotency and differentiation capability of HWJSC and other types of MSC as compared to 2D methods (Hess et al., 2017; Mousavifard et al., 2020; Thakur et al., 2022). The fact that FIBRIAGAR-3D succeeded in improving these markers on cultured HWJSC could contribute to obtaining cell populations with increased differentiation potential and, therefore, with expanded clinical applications. Although the HWJSC differentiation potential has already been applied to the generation of different stromal and epithelial tissues (Garzón et al., 2013; Durand-Herrera et al., 2018; Garzon et al., 2020), the cell populations cultured in FIBRIAGAR-3D may be able to differentiate other types of cells and tissues with potential clinical usefulness. Although the analysis of surface markers of MSC cultured in FIBRIAGAR-3D is compatible with a high differentiation potential, studies analyzing their differentiation capability to the osteogenic, chondrogenic and adipogenic lineages are needed (Nieto-Aguilar et al., 2011).

Another important feature of HWJSC is their very active metabolic activity and their capability to synthetize different ECM molecules such as collagen fibers and basement membrane components (Dan et al., 2017; Garzón et al., 2020; Sánchez-Porras et al., 2023). To determine if cells cultured in the FIBRIAGAR-3D system retain this capability, we evaluated the synthesis of type-I and type-IV collagen, and we found that 3D culturing significantly contributed to increase the percentage of cells actively synthetizing type-I collagen, while the expression of type-IV collagen remained unaltered. Again, these results suggest that 3D culture contributed to improve the metabolic activity of cultured HWJSC or, at least, did not compromise their intrinsic potential to synthetize these components. In general, these results suggest that FIBRIAGAR-3D could contribute to retain the phenotype and metabolic activity of cultured cells, whereas traditional 2D culturing was associated to an alteration of HWJSC markers and a reduction of their biosynthetic activity.

It is well known that HWJSC are immunoevasive, and the clinical use of these cells in patients with different conditions are normally devoid of relevant side effects. In addition, rejection of allogeneic MSC is very slow as compared with other cell types (Ankrum et al., 2014). For this reason, we evaluated if FIBRIAGAR-3D could alter the immunogenic properties of HWJSC cultured in this system. Strikingly, our results showed that cells corresponding to the FA100 group synthetized and secreted the lowest amounts of immune system signaling molecules, with a significant reduction of all the analyzed molecules except IL10. Due to the fact that IL10 is known to exert potent anti-inflammatory effects (Shih et al., 2023), whereas most of the other molecules analyzed here are strong pro-inflammatory factors (Niedźwiedź et al., 2022), our results could imply that FIBRIAGAR-3D is not associated to an increase in the inflammatory potential of cells cultured in this system. The reduced presence of these signaling molecules in the FA100 group could be related to the capability of FIBRIAGAR-3D to reproduce the microenvironment of the native tissues more efficiently than traditional 2D culture systems. In this sense, previous reports demonstrated that the use of 3D culture systems can significantly influence the expression of several interleukins and inflammatory mediators of cells cultured in this systems (Brezulier et al., 2020). However, the presence of a dense network of fibrillar and non-fibrillar components in the biomaterial could also affect the diffusion potential of the biomolecules released by the cultured cells, and we cannot exclude the possibility that part of the released factors could be adsorbed and retained by the biomaterial.

To determine the putative immunogenic effects of cells cultured in the 3D system, we analyzed 8 relevant cell signaling molecules secreted by the cultured cells. As shown in Table 3, cultured cells synthetized several signaling molecules, especially IL6, IL8 and GM-CSF, with differences among study groups. In general, culture of the HWJSC using FA50 tended to show increased amounts of the 8 molecules analyzed, here, whereas FA100 tended to decrease the concentration of these molecules. Specifically, FA100 was associated with a statistically significant decrease of GM-CSF, IFN-g, IL2, IL4, IL6, IL8, and TNFα, although non-significant differences were detected for IL10.

The mechanisms associated to the positive biological effects of FIBRIAGAR-3D have not yet been determined. However, we may hypothesize that this culture platform could offer the cells a more natural microenvironment than 2D culture systems, with numerous adhesion molecules and biomolecular clues able to promote cell physiology and proliferation, without altering their differentiation potential. In fact, it has been previously demonstrated that cell survival and cell growth processes strictly depend on the availability of the cells to adhere and attach to the surrounding ECM (Yan et al., 2014). As compared to classical 2D culture systems, FIBRIAGAR-3D offers the cells the possibility of establishing not only cell-cell interactions, but also cell-matrix interactions able to promote cell physiology, as suggested for cells cultured in other types of biomaterials (Habanjar et al., 2021). In the case of FIBRIAGAR-3D, its positive biological effects could be explained by the presence of abundant bioactive molecules of the human plasma including numerous types of RNA, DNA, lipids, and metabolites (Nevalainen et al., 2021), along with a complex proteasome in which a large number of biofunctional proteins can be found (Choi et al., 2021). Specifically, fibrin hydrogels are known to provide cell binding sites able to promote cell attachment, proliferation and migration within the hydrogel (Ehrbar et al., 2005; Li et al., 2015). In contrast to 2D systems, culturing human cells within biomaterials containing plasma components would offer the cells an array of bioactive molecules able to reproduce the native microenvironment of the cultured cells. In fact, the use of culture media containing the main components of human plasma can efficiently induce physiologic transcriptional responses in human cultured cells (Leney-Greene et al., 2020).

Another possible explanation to the positive effects of FIBRIAGAR-3D could be the related to the biomechanical properties of this biomaterial as compared to 2D systems, as it has been previously demonstrated that the biomechanical properties of biomaterials can significantly influence cell physiology (Brezulier et al., 2020; Liguori et al., 2020). In this regard, we previously found that concentration of the different components of fibrin-agarose hydrogels are significantly associated to the viscoelastic properties of these biomaterials and to the capability of cells to synthetize ECM molecules within the hydrogels (Ionescu et al., 2011; Ionescu et al., 2020). Although additional studies should be carried out, it is likely that the biomechanical properties of the different concentrations of FIBRIAGAR-3D can influence its biological properties, cell-cell adhesion, and physiology when HWJSC are cultured within. In the same sense, it would be necessary to evaluate if the effects of FIBRIAGAR-3D are related to the concentration of cells cultured within this matrix. Although we used the cell concentration that previously allowed us to generate a pre-vascularized oral mucosa substitute by tissue engineering (Blanco-Elices et al., 2021), alternative cell concentrations should also be evaluated.

One interesting question refers to the most adequate concentration of FIBRIAGAR-3D showing the best biological results on HWJSC. In general, we found that the most appropriate results were obtained for FA100 in terms of cell proliferation, viability, phenotype, biosynthetic activity, and synthesis of immune-related molecules. However, the results obtained with lower concentrations of FIBRIAGAR-3D did not reach the effectiveness of FA100 and, in some cases, were significantly worse than the control 2D culture systems. The influence of concentration on the biological properties of biomaterials was demonstrated by numerous previous works (Loukelis et al., 2023; Ortiz-Arrabal et al., 2023). In the case of FIBRIAGAR-3D, the highest concentrations of the fibrin-agarose biomaterial contain the most abundant bioactive factors, making them more bioavailable for the cells cultured within.

Altogether, these results support the use of FIBRIAGAR-3D to improve current culture methods of HWJSC for cell therapy and tissue engineering purposes. Not only this technology is able to increase the number of cells by increasing cell proliferation, but also, the system demonstrated to be safe for the cells, with no induction of cell apoptosis or immunogenicity, and without altering cell metabolism or cell phenotype. As compared to previously described culture systems (Yen et al., 2023), FIBRIAGAR-3D has the advantage of obtaining larger cell populations in a shorter time, with cells showing a more physiological phenotype and, likely, an increased differentiation potential and pro-regenerative effect. However, the clinical application of this system would require adaptation of GMP facilities to 3D culturing, which could be challenging and could require the use of closed automated biofabrication systems (Zhang et al., 2022) and standardization of the concentration of fibrinogen in the plasma used to generate the scaffold. In addition, it is important to note that the cells cultured in FIBRIAGAR-3D are immersed in a three-dimensional scaffold matrix. Although this scaffold containing cells could be directly used in a number of clinical applications using the matrix as a carrier system (Gila-Vilchez et al., 2023), some specific applications may require releasing the cells from the scaffold by enzymatic digestion with plasmin or other fibrinolytic proteins (Lynch et al., 2022), allowing further expansion in 2D culture. Future studies should determine the feasibility of this method for use in tissue engineering, and the potential effects of residual substances that may remain in the cell culture.

The potential usefulness of this 3D culture system should be determined for other types of MSC and other types of human cells that typically proliferate very low in culture, such as the epithelial corneal cells (Chato-Astrain et al., 2021), skin and oral mucosa keratinocytes (Ortiz-Arrabal et al., 2022) and urethral epithelial cells, among others. The potential applications of FIBRIAGAR-3D include not only improving current cell culture methods, but also, generating novel bioartificial tissues by tissue engineering for the clinical treatment of diseases affecting the human cornea (Pellegrini et al., 2018), skin (Egea-Guerrero et al., 2019), oral mucosa (Blanco-Elices et al., 2021) and urethra (Casarin et al., 2022).

## Data Availability

The raw data supporting the conclusion of this article will be made available by the authors, without undue reservation.
