# Supplementary material for: A novel 3D biofabrication strategy to improve cell proliferation and differentiation of human Wharton’s jelly mesenchymal stromal cells for cell therapy and tissue engineering
Source: Front Bioeng Biotechnol. 2023 Aug 10;11:1235161. doi: 10.3389/fbioe.2023.1235161 (PMC10448765; doi:10.3389/fbioe.2023.1235161)

**Supplementary Figure S1**. Analysis of cells undergoing apoptosis as determined by the TUNEL method. Illustrative images are shown for HWJSC cultured on culture plates (CTR-2D) and in the 25%, 50%, 75% and 100% concentrations of FIBRAGAR-3D (FA25, FA50, FA75 and FA100, respectively). Scale bars: 50µm. The histogram shows the average and standard deviation percentage of positive cells found in each study group.


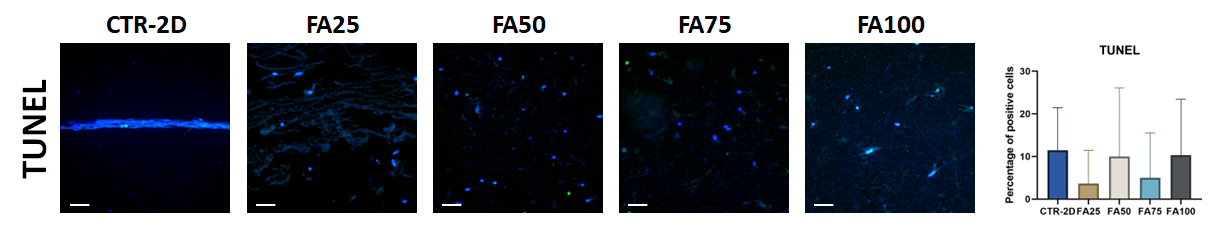

Supplement: Supplementary file 1 [file Table1.DOCX]
