# Supplementary material for: A novel 3D biofabrication strategy to improve cell proliferation and differentiation of human Wharton’s jelly mesenchymal stromal cells for cell therapy and tissue engineering
Source: Front Bioeng Biotechnol. 2023 Aug 10;11:1235161. doi: 10.3389/fbioe.2023.1235161 (PMC10448765; doi:10.3389/fbioe.2023.1235161)

**Supplementary Figure S2**. Control samples used for the different immunohistochemical and TUNEL analyses carried out in this work. For the immunohistochemical analyses, native human controls showing positive and negative signal (CTR+ and CTR-, respectively) and study samples in which the staining method was performed without primary antibody (TEC CTR) were used as technical controls. For TUNEL, CTR+ and CTR- controls are shown. Scale bars: 50µm.


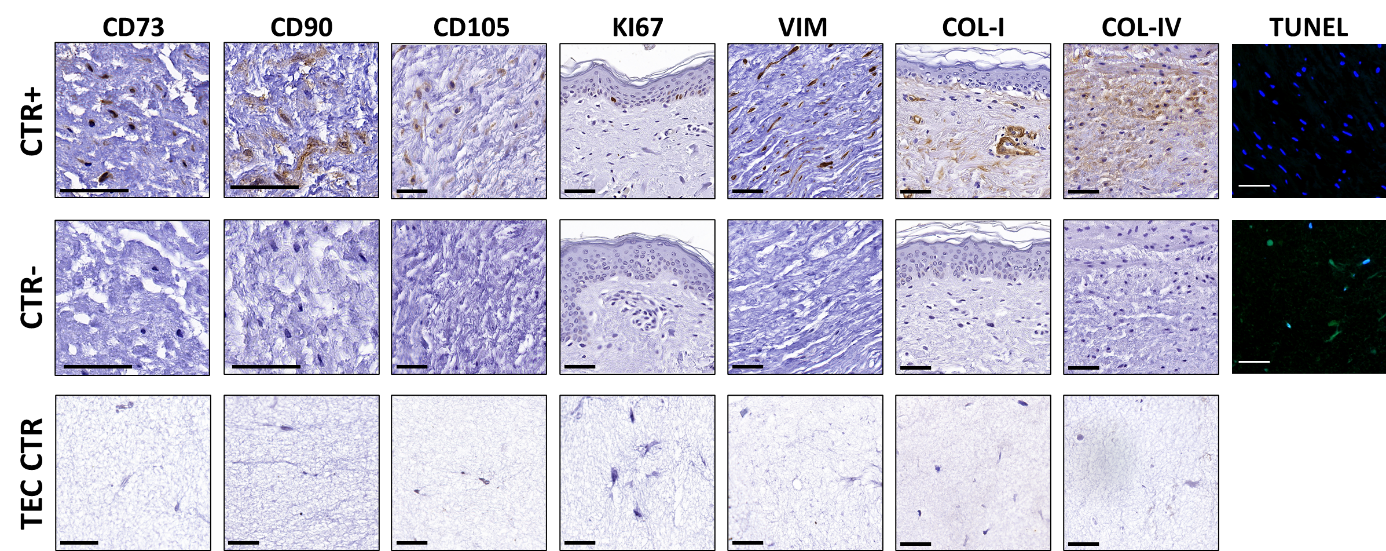

Supplement: Supplementary file 2 [file Table2.DOCX]
